# Sexuality Education in Schools

**DOI:** 10.1055/s-0038-1676776

**Published:** 2018-12

**Authors:** Brazilian Committee Specialized in Sexology of FEBRASGO, São Paulo, Brazil, Brazilian Association of Studies on Human Sexuality (SBRASH), São Paulo, Brazil

The Brazilian Committee Specialized in Sexology of the Brazilian Federation of Gynecology and Obstetrics Associations (FEBRASGO) and the Brazilian Association of Studies on Human Sexuality (SBRASH) have been asked to talk about the controversy involving sexuality education (SE) in elementary and high schools. The broad discussion of the members of the FEBRASGO committee and the board of SBRASH, that is, physicians (gynecologists, obstetricians, psychiatrists and urologists), psychologists, and educators, resulted in the present editorial.

According to the World Health Organization (WHO), SE should be a comprehensive educational process on sexuality, comprising knowledge, skills, and values that enable young people to make informed and responsible choices related to their sexual experiences. Sexuality education offers opportunities to explore and build one's own values, behaviors, and attitudes to acquire decision-making skills, improve communication skills, and reduce behaviors that represent sexual and general risks.^1^

Sexuality is a central dimension of life that is fully experienced in adolescence, a period marked by impulsivity, experimentation, restlessness, and by reduced concern toward prevention aspects.^1^ Therefore, it is imperative to incorporate sexuality into the pedagogical process of the school system to complement the role of the family in the construction of one's sexuality. Our position is also based on the Declaration of Sexual Rights of the World Association for Sexual Health,^2^ which considers that everyone shall have:

9. The right to information – [...] access to scientifically accurate and understandable information related to sexuality, sexual health, and sexual rights through diverse sources. Such information should not be arbitrarily censored, withheld, or intentionally misrepresented.

10. The right to education and the right to comprehensive sexuality education – [...] Comprehensive sexuality education must be age appropriate, scientifically accurate, culturally competent, and grounded in human rights, gender equality, and a positive approach to sexuality and pleasure.

The development of SE programs in schools face barriers that prevent their effectiveness.^3^ ^4^ Following this perspective, UNESCO has evaluated the impact of SE programs by reviewing 87 studies on their effectiveness and concluded that sexuality education:

- does NOT increase sexual activity in adolescence,
- does NOT encourage sexual risk-taking behavior, and
- does NOT increase the risk for sexually transmitted infections (STIs).

Many of these studies show SE helps postpone sexual initiation and reduce the number of sexual partners, and that it is effective in increasing the use of condoms and contraceptive methods.^5^ A cost-effectiveness evaluation of these programs showed government savings of US$ 67,825.00 in a European country per individual who did not have HIV due to these SE programs.

In 2010, UNESCO revised and issued its International Technical Guidance on Sexuality Education to all countries of the world, recommending nations to address topics related to sexuality in school education, with guidelines to conduct this task with children of at least 5 years old, adolescents and adults of all ages (Table 1).^5^

**Table 1:** Principles of sexuality education according to UNESCO^5^

Sexuality education conducted in formal and non-formal contexts should observe the following principles:
✓ Be based on scientific evidence
✓ Be comprehensive (increment information about the theme)
✓ Be appropriate to the child's age and cognitive development
✓ Be specific to each gender
✓ Be culturally relevant and transformative

**Source:** UNESCO. EVALUATION OF SEXUALITY EDUCATION; Goal of Sexuality Education. Berlin: United Nations Education, Scientific and Cultural Organization; 2017.

In order to achieve the standard set by UNESCO, combined education and health actions are required, involving training to parents and elementary and high school teachers, who may feel uncomfortable teaching sexuality topics when the content is in conflict with their cultural values and beliefs. Health professionals should have scientific knowledge about sexuality and define strategies based on scientific evidence. This implies that the didactic material for sexuality education in schools needs to be analyzed and validated by health and education professionals together. And these professionals should get acquainted with the results of scientific studies on the subject and the principles set by UNESCO to enhance the quality of SE programs (Table 1).^5^

The American College of Obstetricians and Gynecologists (ACOG) advocates the participation of gynecologists in sexuality education programs because of the broad access these professionals have to important aspects of adolescent sexuality and the sexual health of couples.^6^ In Brazil, sexology is one of the areas in which gynecologists and obstetricians (GO) operate, so the Brazilian Committee Specialized in Sexology of FEBRASGO is responsible for providing didactic material based on scientific evidence and for participating in sexuality education programs and in those that provide support to people with sexual dysfunctions. FEBRASGO grants sexologist degrees through an annual test, which evaluates the knowledge on sexuality of gynecologists and obstetricians during the medical residency program in Gynecology and Obstetrics or in an equivalent program. To apply for a sexologist degree, the physician needs a prior GO specialist degree (TEGO).

The Brazilian Association of Studies on Human Sexuality (SBRASH) grants human sexuality specialist degrees (TESH) in the fields of Sexual Therapy, Sexual Education, and Social Sexology through a test held during SBRASH biennial congresses.

The Brazilian Federation of Gynecology and Obstetrics Associations (FEBRASGO) and SBRASH both recognize sexuality education should be incorporated into the syllabuses of elementary and high schools to lower the rates of sexual violence (sexual abuse and rape) and reduce risk-taking behaviors in adolescence.^1^ The road toward a sexually healthy society requires a serious approach to the subject, scientific evidence, and ethical responsibility. Hence, professionals responsible for SE in schools in Brazil, with the legal support from the National Curricular Parameters (BRAZIL, 1998) should seek training provided by entities specialized in human sexuality, such as FEBRASGO and SBRASH, and be committed to employing evidence-based methodologies.
